# Methods to perform systematic reviews of patient preferences: a literature survey

**DOI:** 10.1186/s12874-017-0448-8

**Published:** 2017-12-11

**Authors:** Tsung Yu, Nomin Enkh-Amgalan, Ganchimeg Zorigt

**Affiliations:** Department of Public Health, China Medical University, No 91 Hsueh-Shih Road, Taichung, Taiwan

**Keywords:** Patient preference, Systematic review, Meta-analysis

## Abstract

**Background:**

Systematic reviews are a commonly used research design in the medical field to synthesize study findings. At present—although several systematic reviews of patient preference studies are published—there is no clear guidance available for researchers to conduct this type of systematic review. The aim of our study was to learn the most current practice of conducting these systematic reviews by conducting a survey of the literature regarding reviews of quantitative patient preference studies.

**Methods:**

Our survey included systematic reviews of studies that used a stated quantitative preference design to elicit patient preferences. We identified eligible reviews through a search of the PubMed database. Two investigators with knowledge of the design of patient preference studies independently screened the titles and abstracts, and where needed, screened the full-text of the reviews to determine eligibility. We developed and pilot-tested a form to extract data on the methods used in each systematic review.

**Results:**

Our search and screening identified 29 eligible reviews. A large proportion of the reviews (19/29, 66%) were published in 2014 or after; among them, nine reviews were published in 2016. The median number of databases searched for preference studies was four (interquartile range = 2 to 7). We found that less than half of the reviews (13/29, 45%) clearly reported assessing risk of bias or the methodological quality of the included preference studies; not a single review was able to perform quantitative synthesis (meta-analysis) of the data on patient preferences.

**Conclusion:**

These results suggest that several methodological issues of performing systematic reviews of patient preferences are not yet fully addressed by research and that the methodology may require future development.

## Background

Making healthcare decisions is difficult since multi-dimensional factors are often involved. For example, when patients are in the situation to choose treatments for their conditions, they may need to jointly consider factors such as the benefits, harms, costs and inconveniences of each treatment in order to choose their most “preferred” option. Some patients are more concerned about the side effects of treatments; others may be more risk-tolerant. Thus, learning about patient preference information—or the “assessments of the relative desirability or acceptability to patients of specified alternatives” [1]—is critical in making an informed and patient-centered decision. It becomes even more important when multiple options exist, especially when it is unclear which option is superior or when the preferences vary considerably between patients [2].

Many regulatory agencies and health technology assessment bodies have also recognized the importance of studying patient preferences to improve their decision-making [3, 4]. Patient preference information can be elicited in qualitative studies such as patient interviews and focus groups. Or, it can be elicited quantitatively from a population of patients using the “stated preference” approaches developed mostly in the health economics field, including designs such as the rating scale, standard gamble, time trade-off or discrete choice experiment [5, 6]. In the end, individual patients may make their choices based on their own circumstances (socio-demographics, disease severity, comorbidities, or financial situations) and their own preferences. However, to make decisions such as drug approvals or reimbursement at a population level, preferences elicited from a survey of population can be beneficial to the decision-making for the entire population [7].

A systematic approach to studying patient preferences seems valuable. First, we need to conduct more preference-eliciting surveys and then do it across different populations to capture the heterogeneity, if such heterogeneity exists. When multiple preference-eliciting studies are done, a systematic review of these studies may then be needed to synthesize and summarize the study findings. Although several systematic reviews of patient preference studies are published, at present no standard guidance is available yet for researchers to conduct this type of systematic review.

Recently some efforts have been devoted to developing these review methodologies. For example, Yepes-Nuñez et al. [8] have done a systematic survey of patient preference reviews to identify items that previous reviewers have used in making risk of bias assessment of primary studies addressing preferences. They then grouped these items into seven domains for assessing the risk of bias, such as the instrument health state presentation. Besides risk of bias assessment, review methodologies for searches of preference studies, or qualitative and quantitative synthesis of primary study results are also highly needed from reviewers to conduct a preference review, which would be much different from that for a regular intervention review. Therefore, our goal was to gain an overview of the most current methods and practice that have been reported in the literature for conducting systematic reviews of quantitative patient preference studies.

## Methods

### Selection of studies

Our approach was not to perform a full systematic review of reviews, but rather to do a literature survey of systematic reviews. We focused on the methodology used by reviewers to conduct research synthesis of evidence on quantitative patient preferences. We restricted our selection of systematic reviews to the PubMed database. We adapted existing search filters [9, 10] and developed a strategy for the PubMed database with two concepts for the search: “systematic review” and “patient preferences” (*(search[tiab] OR meta-analysis[pt] OR MEDLINE[tiab] OR (systematic[tiab] AND review[tiab])) AND (“patient preference”[mh] OR preference[ti] OR preferences[ti])*). The search was performed on December 1, 2016.

Our pre-specified study inclusion criterion was any systematic review that aimed to synthesize quantitative evidence of patient preferences for attributes of an intervention or a health technology. This included medications, surgeries, medical devices, behavioral interventions, diagnostic tests and screening programs. The eligible systematic review had to include some studies using a quantitative stated preference design to assess patient preferences (such as the rating scale, visual analogue scale, standard gamble, time trade-off, contingent valuation, discrete choice experiment, or best-worst scaling). We excluded reviews that focused only on qualitative research. In some preference studies, participants were only asked to make choices between alternatives (for example, Drug A vs. Drug B) but the attributes of these alternatives (for example, benefits, harms, costs, and inconveniences) were not explicitly stated. We did not include reviews that focused only on this type of studies since they did not generate evidence on quantitative patient preferences for any attributes of an intervention or a health technology. Only English articles were included in our sample.

Two investigators with knowledge on the design of patient preference studies independently screened the list of search results to assess study eligibility by reviewing titles and abstracts. Where the reviewers could not make decisions on eligibility based on titles and abstracts alone, the full-text of each systematic review was downloaded and examined. Disagreement on the study eligibility was resolved through group discussion.

### Data extraction and analysis

We developed and pilot-tested a data extraction form for this survey. The main items in the form included the following: objectives and conclusions of the systematic review, the criteria used to include/exclude patient preference studies, the methods used to search for preference studies, the methods used to assess the quality of preference studies, and the qualitative or quantitative methods used to synthesize study findings. One experienced reviewer independently performed the data extraction and assessment of methodology; another reviewer reviewed and checked the answers to each item in the form. Disagreement between the two reviewers was resolved by group discussion. We summarized these results in the tables of study characteristics.

## Results

### Search results

**Fig. 1:**
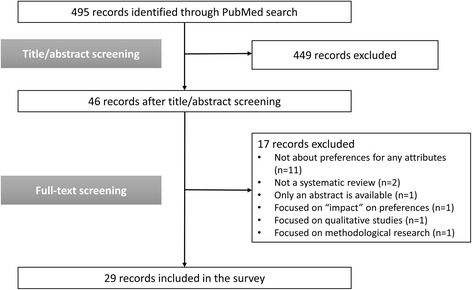
Study flow

We identified 495 records from the search of the PubMed database. After title/abstract screening, 449 records were excluded, leaving 46 records. We excluded 17 records after full-text screening; the reasons for exclusion can be found in the Fig. 1. Most of the records were excluded because they focused on studies that assessed the preferences for alternatives (e.g., Drug A vs. Drug B) but the attributes of these alternatives were not explicitly stated in their assessment. Finally, we included 29 records (systematic reviews) in this literature survey.

### Characteristics of included reviews

Table 1 lists the 29 included systematic reviews [10–38]. The number of . About two-thirds (19/29) of the reviews were published in 2014 or after; among them, 9 reviews were published in 2016. About half of the included reviews were on cancers (14/29); three were on psoriasis; three were on type 2 diabetes; two were on attention-deficit/hyperactivity disorder, and the remaining seven reviews were each about different diseases. Among reviews on cancers (*n* = 14), 11 were studying the preferences for cancer treatment and three were studying preferences for cancer screening.

**Table 1:** Included systematic reviews

First author	Year	Title	Objective	Date of search
Belinchón [11]	2016	Adherence, satisfaction & preferences for treatment in patients with psoriasis in the European Union: a systematic review of the literature	“To examine & describe the current literature on patient preferences, satisfaction & adherence to treatment for psoriasis in the European Union.”	Dec. 2014
Bereza [12]	2015	Patient preferences in severe COPD & asthma: a comprehensive literature review	“To summarize original research articles determining patient preference in moderate-to-severe disease.” [COPD & asthma]	Nov. 2014
Blanchard [13]	2016	Assessing head & neck cancer patient preferences & expectations: a systematic review	“We conducted a systematic review of the current evidence regarding the preferences & priorities of patients with head & neck cancer.”	Sept. 2016
Blinman [14]	2010	Patients’ preferences for chemotherapy in non-small-cell lung cancer: a systematic review	“To find, evaluate & summarise studies quantifying the survival benefits that cancer patients judged sufficient to make chemotherapy for NSCLC worthwhile.”	2009
Bradley [15]	2007	Review of patterns of practice & patients’ preferences in the treatment of bone metastases with palliative radiotherapy	“To review the patterns of practice among radiation oncologists & patients’ preferences in the treatment of bone metastases with palliative radiotherapy.”	June 2006
Brooker [16]	2013	Quantitative patient preference evidence for health technology assessment: a case study	“To explore the feasibility & desirability of incorporating patient preferences within the health technology assessment process by working through a case study.” [COPD]	March 2011
Currie [17]	2014	A systematic review of patient preference elicitation methods in the treatment of colorectal cancer	“To assess the use of patient preference in colorectal cancer treatment.”	March 2014
Damm [18]	2014	Preferences of colorectal cancer patients for treatment & decision-making: a systematic literature review	“To identify the preferences of CRC patients with regard to treatment preferences & involvement in the decision-making process regarding treatment choices.”	Sept. 2012
Eek [19]	2016	Patient-reported preferences for oral vs. intravenous administration for the treatment of cancer: a review of the literature	“To evaluate the administration preferences of cancer patients, specifically between oral & intravenous treatment, as well as the factors contributing to preference.”	Jan. 2015
Eiring [20]	2015	What matters to patients? A systematic review of preferences for medication associated outcomes in mental disorders	“To investigate patients’ preferences for outcomes associated with psychoactive medications.”	Sept. 2013
Emberton [21]	2010	Medical treatment of benign prostatic hyperplasia: physician & patient preferences & satisfaction	“This review evaluates patients’ & physicians’ preferred treatment options for managing BPH & patient satisfaction with therapy.”	June 2008
Gutknecht [22]	2016	A systematic review on methods used to evaluate patient preferences in psoriasis treatments	“To give an overview of methods that have been used in international published studies to evaluate patient preferences in psoriasis treatments.”	Dec. 2014
Hamelinck [23]	2014	Patients’ preferences for surgical & adjuvant systemic treatment in early breast cancer: a systematic review	“To give an overview of patient self-reported factors affecting preferences for breast conserving surgery vs. mastectomy, the minimal benefit patients require from adjuvant chemotherapy or adjuvant hormonal therapy to consider it worthwhile, & factors influencing this minimally-required benefit.”	Oct. 2012
Joy [24]	2013	Patient preferences for the treatment of type 2 diabetes: a scoping review	“To identify & assess the literature reporting on the treatment preferences of adult patients with type 2 diabetes.”	Jan. 2013
Lytvyn [25]	2016	Patient values & preferences on trans-catheter or surgical aortic valve replacement therapy for aortic stenosis: a systematic review	“To investigate patients’ values & preferences regarding aortic valve replacement therapy for aortic stenosis.”	Unclear
MacLean [26]	2012	Patient values & preferences in decision making for antithrombotic therapy: a systematic review	“We conducted a systematic review relating to values & preferences of patients considering antithrombotic therapy.”	Sept. 2009
Mansfield [27]	2016	Stated preference for cancer screening: a systematic review of the literature, 1990–2013	“We reviewed stated-preference studies for breast, cervical, & colorectal cancer screening to identify the types of attributes included, the use of questions to assess uptake, & whether gaps exist in these areas.”	July 2013
Phillips [28]	2006	A review of studies examining stated preferences for cancer screening	“To conduct a systematic review of stated preference studies for cancer screening, identify gaps in the literature, & determine which types of research should be conducted in the future.”	May 2005
Purnell [29]	2014	Patient preferences for noninsulin diabetes medications: a systematic review	“To systematically review patient preferences for noninsulin diabetes medications in adults with type 2 diabetes.”	Jan. 2013
Sadique [30]	2011	Women’s preferences regarding options for management of atypical, borderline or low-grade cervical cytological abnormalities: a review of the evidence	“To identify empirical studies evaluating women’s preferences regarding alternative management pathways & to compare the impact of alternative elicitation methods on results.”	March 2009
Schatz [31]	2015	Systematic review of patients’ & parents’ preferences for ADHD treatment options & processes of care	“To synthesize reports across existing DCE, BWS, TTO, & SGI studies to assess which aspects of ADHD treatment are most studied as well as most preferred & influential in treatment decisions.”	Oct. 2014
Schmidt [32]	2016	Preferences of lung cancer patients for treatment & decision-making: a systematic literature review	“To identify the preferences of lung cancer patients with regard to their treatment & involvement in the decision-making process.”	Sept. 2012
Showalter [33]	2015	Factors that influence patient preferences for prostate cancer management options: a systematic review	“We performed a systematic review to evaluate evidence regarding factors that influence patient preferences for management options for localized prostate cancer.”	April 2014
Stewart [34]	2016	Preference for pharmaceutical formulation & treatment process attributes	“To examine studies on preferences for pharmaceutical treatment process attributes, focusing on research in diabetes, oncology, osteoporosis, & autoimmune disorders.”	Oct. 2013
Umar [35]	2012	Elicitation & use of patients’ preferences in the treatment of psoriasis: a systematic review	“To critically review the scientific evidence regarding the elicitation & use of patients’ preferences in psoriasis treatment.”	Nov. 2009
Van Brunt [10]	2011	Preferences related to attention-deficit/hyperactivity disorder & its treatment	“To identify & summarize published research on preferences related ADHD & its treatment, while suggesting directions for future research.”	Unclear
Von Arx [36]	2014	The patient perspective of diabetes care: a systematic review of stated preference research	“To examine how stated preference methods are applied in diabetes care, & to evaluate the value of this information in developing the patient perspective in clinical & policy decisions.”	May 2013
Wilke [37]	2016	Patient preferences for oral anti- coagulation therapy in atrial fibrillation: a systematic literature review	“To systematically analyse the scientific literature assessing the preferences of AF patients with regard to long-term oral anticoagulant treatment.”	2015
Wortley [38]	2014	Assessing stated preferences for colorectal cancer screening: a critical systematic review of discrete choice experiments	“To undertake a systematic review of discrete choice experiments of CRC screening.”	April 2013

*ADHD* Attention-deficit/hyperactivity disorder, *AF* Atrial fibrillation, *BPH* Benign prostatic hyperplasia, *BWS* Best-worst scaling, *COPD* Chronic obstructive pulmonary disease, *CRC* Colorectal cancer, *DCE* Discrete choice experiment, *NSCLC* Non-small-cell lung cancer, *SGI* Standard gamble interview, *TTO* Time trade-off

Table 2 shows the characteristics of these 29 included reviews. The median number of databases searched was four (interquartile range = 2 to 7). Six reviews (6/29, 21%) searched one database; four reviews (4/29, 14%) searched two; eleven reviews (11/29, 38%) searched three to five and eight reviews (8/29, 28%) searched more than five. All reviews (*n* = 29) searched for studies through PubMed or MEDLINE, 19/29 (66%) through EMBASE, 9/29 (31%) through CINAHL, 9/29 (31%) through PsycINFO, and 8/29 (28%) through EconLit. Almost half of the reviews (14/29, 48%) searched reference lists of the included studies. Few reviews searched for non-English articles (7/29, 24%) or for conference abstract/unpublished studies/grey literature (6/29, 21%). The search strategies used in each review to identify studies of patient preferences are summarized in Table 3. The terms developed by these reviews are focused mainly on “patient preferences” and the name of the study design used to elicit patient preferences.

**Table 2:** Characteristics of the systematic reviews

Characteristics of the systematic review (*N* = 29)	
Number of databases searched	
*One, n (%)*	6 (21)
*Two, n (%)*	4 (14)
*Three to five, n (%)*	11 (38)
*More than five, n (%)*	8 (28)
Database searched	
*PubMed or MEDLINE, n (%)*	29 (100)
*EMBASE, n (%)*	19 (66)
*CINAHL, n (%)*	9 (31)
*PsycINFO, n (%)*	9 (31)
*EconLit, n (%)*	8 (28)
*Other databases, n (%)*	14 (48)
Searched for non-English articles, n (%)	7 (24)
Reported searching reference lists of included studies, n (%)	14 (48)
Reported searching for conference abstracts, unpublished studies or grey literature, n (%)	6 (21)
Reported methods used to assess study eligibility, n (%)	25 (86)
Reported assessing risk of bias or methodological quality of included studies, n (%)	13 (45)
Used quantitative methods (meta-analysis) to synthesize study data, n (%)	0 (0)
Funding sources listed	
*No funding, n (%)*	3 (10)
*Government, n (%)*	14 (48)
*Industry, n (%)*	9 (31)
*Others, n (%)*	5 (17)
*Unclear, n (%)*	2 (7)

**Table 3:** Search strategies used for patient preferences

Review	Strategy used to search for preference studies
Belinchón, 2016	Search terms included “preferences” and “utility”.
Bereza, 2015	Search terms included “preference” and “utilities”.
Blanchard, 2016	Search terms included “patient preference”, “patient priorities” and the different patient stated-preference methods, such as “rating”, “ranking”, “best-worst”, “self-explicated”, “value-based conjoint analysis”, “rating-based conjoint analysis”, “choice-based conjoint analysis”, “take it or leave it”, “tradeoff” and “trade-off”.
Blinman, 2010	Search terms included “preference”, “utility”, “attitude”, “expectation” and “willingness”.
Bradley, 2007	Search terms included “patient preferences”, “patient satisfaction” and “patient participation”.
Brooker, 2013	Search terms included those that relate to “patient perspectives”, “satisfaction”, “preferences” and “values”.
Currie, 2014	Search terms included those that relate to existing patient preference elicitation methodologies: “patient preference”, “shared decision-making”, “patient involvement”, “patient participation”, “patient satisfaction”, “physician–patient relation”, “standard gamble”, “time trade-off”, “willingness to trade”, “willingness to pay”, “decision board” and “discrete choice experiment”.
Damm, 2014	Search terms included “patient” and “preference or willingness”.
Eek, 2016	Search terms included “preference”, “prefer”, “preferred”, “choice”, “select” and “selection”.
Eiring, 2015	Comprehensive search strategies were developed (see http://bmjopen.bmj.com/content/5/4/e007848).
Emberton, 2010	Search terms included “patient preference”, “perception” and “satisfaction”.
Gutknecht, 2016	Search terms included keywords of preference methods in health economics: “preferences”, “conjoint analysis”, “choice model”, “discrete choice”, “DCE”, “decision analysis”, “multi-criteria decision analysis”, “MCDA”, “multi-attribute utility”, “analytic hierarchy process”, “AHP”, “trade-off”, “best-worst scaling”, “willingness-to-pay”, “WTP”, “willingness to accept”, “contingent valuation” and “standard gamble”.
Hamelinck, 2014	Search terms included “patient preference”, “choice”, “decision”, “choice behavior”, “decision making” and “patient satisfaction”.
Joy, 2013	Search terms included “conjoint analysis”, “satisfaction”, “choice model”, “stated preference”, “discrete choice”, “DCE”, “decision analysis”, “preferences”, “multicriteria decision analysis”, “MCDA”, “multi-attribute utility”, “analytic hierarchy process”, “trade off”, “self-explicated”, “best-worst scaling”, “utilities”, “preference weight”, “willingness to pay”, “WTP”, “willingness to accept”, “contingent valuation”, “priorities” and “valuation”.
Lytvyn, 2016	Search terms included “health utility”, “patient values”, “patient preferences” and “health-related quality of life”.
MacLean, 2012	Unclear
Mansfield, 2016	Search terms included “conjoint analysis”, “discrete choice”, “discrete ranking” and “discrete rank”.
Phillips, 2006	Search terms included “patient satisfaction”, “numerical data”, “consumer satisfaction”, “health knowledge”, “attitudes”, “practice”, “choice behavior”, “conjoint analysis”, “contingent valuation”, “stated preference”, “discrete choice” and “willingness to pay”.
Purnell, 2014	Search terms included methods to assess patient preferences (e.g., “conjoint analysis”, “decision analysis”, “utilities”, and “stated preferences”).
Sadique, 2011	Search terms included “preferences”, “values”, “willingness-to-pay” and “utility”.
Schatz, 2015	Search terms included “patient preferences”, “stated preferences”, “discrete choice”, “conjoint analysis”, “best worst”, “maximum difference”, “standard gamble”, “time trade-off” and “utility values”.
Schmidt, 2016	Search terms included “patient”, “preference”, and “willingness”.
Showalter, 2015	Search terms included “conjoint analysis”, “satisfaction”, “choice model”, “stated preference”, “discrete choice”, “DCE”, “decision analysis”, “preferences”, “multi-criteria decision analysis”, “MCDA”, “multi-attribute utility”, “analytic hierarchy process”, “trade off”, “self-explicated”, “best-worst scaling”, “utilities”, “preference weight”, “willingness to pay”, “WTP”, “willingness to accept”, “contingent valuation”, “priorities” and “valuation”.
Stewart, 2016	Search terms included “stated preference(s)”, “time trade-off”, “standard gamble”, “conjoint”, “contingent valuation”, “discrete choice” and “willingness-to-pay”.
Umar, 2012	Search terms included “patient preferences”, “shared decision-making”, “patient involvement”, “patient participation”, “patient satisfaction” and “physician-patient relation”.
Van Brunt, 2011	Search terms included “health state utility”, “utility”, “discrete choice”, “standard gamble”, “time trade-off”, “quality-adjusted life year”, “conjoint analysis”, “patient preference”, “preference”, “prefer”, “satisfaction”, “acceptability”, “decision” and “choice”.
Von Arx, 2014	Search terms included “stated preference”, “willingness to pay”, “willingness to accept”, “choice modelling”, “conjoint analysis”, “discrete choice experiment” and “contingent valuation”.
Wilke, 2016	Search terms included “discrete choice experiment”, “treatment preference”, “conjoint” and “trade off”.
Wortley, 2014	Search terms included “stated preference” and “choice experiment”.

Most reviews (25/29, 86%) documented the methods used to assess study eligibility, for example, by mentioning the double and independent screening of title/abstract/full-text. Less than half of the reviews (13/29, 45%) clearly reported assessing the risk of bias or the methodological quality of the included preference studies. None of the reviews were able to perform quantitative synthesis (meta-analysis) of the preference data. The types of patient preference study design included in each review—such as the rating scale, visual analogue scale, standard gamble, time trade-off, contingent valuation, discrete choice experiment, and best-worst scaling—are listed in Table 4. A wide range of preference study designs is often included in this type of systematic reviews. Almost half of the reviews (14/29, 48%) were fully or partly funded by government, and 9/29 (31%) were funded by industry (see Table 2).

**Table 4:** Types of included preference studies

Review	Type of included preference studies reported by the review
Belinchón, 2016	CA, DCE
Bereza, 2015	WTP
Blanchard, 2016	RS, SG, TTO, VAS
Blinman, 2010	Preference study design not specified
Bradley, 2007	Preference study design not specified
Brooker, 2013	Preference study design not specified
Currie, 2014	DCE, SG, TTO
Damm, 2014	CA, DCE, RS, SG, TTO
Eek, 2016	CA
Eiring, 2015	CA, DCE, PC, RS, SG, TTO, VAS, WTP
Emberton, 2010	DCE, RS
Gutknecht, 2016	CA, DCE, RS, SG, TTO, VAS, WTP
Hamelinck, 2014	Preference study design not specified
Joy, 2013	CA, CV, RS, SG, TTO, WTP
Lytvyn, 2016	SG
MacLean, 2012	PTOT, RS, SG, TTO, VAS
Mansfield, 2016	CA, DCE
Phillips, 2006	CA, CV
Purnell, 2014	CA, DCE, RS, SG, TTO, VAS, WTP
Sadique, 2011	SG, WTP
Schatz, 2015	BWS, DCE, SG
Schmidt, 2016	CV, DCE, RS, WTP
Showalter, 2015	DCE, TTO
Stewart, 2016	BWS, CV, DCE, SG, TTO, WTP
Umar, 2012	DCE, PTO, SG, TTO, VAS, WTP
Van Brunt, 2011	DCE, SG
Von Arx, 2014	CV, DCE
Wilke, 2016	CA, DCE, PTO, SG, TTO, VAS
Wortley, 2014	DCE

*BWS* Best-worst scaling, *CA* Conjoint analysis, *CV* Contingent valuation, *DCE* Discrete choice experiment, *PC* Pairwise comparison, *PTO* Person trade-off, *PTOT* Probability trade-off technique, *RS* Ranking or rating scale, *SG* Standard gamble, *TTO* Time trade-off, *VAS* Visual analogue scale, *WTP* Willingness to pay

## Discussion

Our survey of the medical literature shows that there has been an increase in systematic reviews on patient preferences being published since 2014. This may suggest an increasing interest in conducting preference-eliciting studies, as well as systematic reviews of these studies. Almost half of the included reviews were on cancers, suggesting the strong interest from cancer researchers or their need to synthesize patient preference data. Although a number of this type of systematic reviews are published, many methodological issues regarding performing these systematic reviews are not yet fully addressed by previous research. For example, there is a lack of clear guidance or consensus on the approaches to searching for preference studies, to assessing methodological quality of preference studies, or to quantitatively synthesizing the data from preference studies.

Systematic reviews are a commonly used research design in the medical field to synthesize study findings—such as the treatment’s benefits, harms or costs—in order to inform evidence-based decision-making. Not only can systematic reviews summarize study results, they can also serve as a way to examine the heterogeneity in different studies or to identify the subgroups for which their results differ from others. There is a potentially large heterogeneity in the findings across patient preference studies, even for those addressing similar questions, because different researchers may use different preference designs, include different items for patients to assign preferences, and on top of that, socio-demographics are known to have great influences on preferences [39, 40]. Therefore, in many situations, systematic reviews may be a useful and necessary way to not only study patient preferences, but at the same time to also deal with these very heterogeneous studies, which poses great challenges to systematic reviewers.

Building up a comprehensive search strategy is one of the most important steps to conducting a systematic review. A comprehensive search strategy will identify as far as possible all relevant studies addressing the research question of interest, which can involve searching in different electronic databases, searching reference lists of included studies, searching for non-English articles, and searching for conference abstracts, unpublished studies or grey literature.

In our survey, most reviews had searched in databases beyond the PubMed/ MEDLINE database (median number of databases searched: 4), but only one-fifth to one-fourth of the reviews had searched for non-English articles (24%) or had searched for conference abstracts, unpublished studies or grey literature (21%). A few methodological questions regarding the search for patient preference studies are still not answered and deserve our attention. For example, are some of the databases such as EconLit (a database focusing on economics publications) required for searching for patient preference studies? Are preference data reported in conference abstracts useful to systematic reviewers? Is searching for non-English articles important? We need more methodological research to address these questions in order to support us in doing a comprehensive search, while at the same time not being lost in the literature. Also, we found that there was a wide range of search terms used by systematic reviewers to retrieve patient preference studies. This indicates the variety of terms used by most investigators to describe patient preferences and a need for search filters. Some groups have developed search filters for preference studies [41, 42], and more research on these search filters is needed to test for their validity.

Another issue we identified in our survey of reviews was that less than half of the studies reported assessing risk of bias or the methodological quality of patient preference studies. Methods to assess study quality/risk of bias of randomized controlled trials, for example, are mature because lots of research efforts have been dedicated to developing assessment tools for this. In our survey, some reviewers such as Purnell and colleagues adapted other existing tools or constructed checklists themselves when they were appraising the study quality [29]. They assessed whether the preference study had properly addressed purposes, respondents, explanation, findings, and significance. Others such as the U.S. Food and Drug Administration also listed several quality standards for patient preference studies in one of their guides [2] and researchers from McMaster University have generated 23 items for assessing the risk of bias in preference studies [8]. Implementing the methodological quality assessment tools for patient preference studies in actual practice would be challenging since there are so many different types of study designs available. We need more research to develop a standard way that can be used by most systematic reviewers to appraise the quality of preference studies.

In a systematic review of randomized controlled trials, meta-analyses (quantitative synthesis) are performed to synthesize data in order to generate more precise estimates. When it is difficult or not reasonable to combine study data, systematic reviewers may choose not to perform a meta-analysis. We found in our survey that no review was able to perform a meta-analysis of patient preferences, because for instance some preference studies may use a visual analogue scale approach and report preference weight ranging from zero to a hundred; some studies may conduct a discrete choice experiment and report coefficients from a conditional logistic regression model. Moreover, these studies may include very heterogeneous patient populations and the items included for preference assessment can vary greatly from study to study. We noticed that there are a few reviews using tables to summarize the preference rankings of the attributes obtained within each study, which helps authors better communicate with readers [29, 36]. Perhaps statistics such as the frequency of an item being ranked as most important could be used as a measure in meta-analysis to synthesize preference data. We need more statisticians and methodologists in this field to develop novel approaches to doing meta-analysis in preference research.

A reporting guideline for patient preference studies is likely to substantially improve the reporting of such studies. We found that systematic reviewers in our survey often needed to include terms such as “utility,” “attitude,” “expectation,” “willingness,” “satisfaction,” and “value” to capture relevant publications, indicating that the definitions and terms used by most investigators for patient preferences may be at present quite inconsistent. Additionally, in several reviews we found that their included studies did not refer to themselves as the specific design for preference-eliciting research (such as visual analogue scale, time trade-off, or discrete choice experiment); instead the investigators described the studies as a cross-sectional survey or patient questionnaire research, which made it more difficult for systematic reviewers to conduct the search. Reviewers rely heavily on the reporting of published articles to perform assessment and synthesis of studies. Only if there is a complete, clear and transparent reporting of the original articles can most systematic reviewer conduct proper evidence synthesis [43]. Similar to having other reporting guidelines, having a guideline on reporting of patient preference studies may indirectly improve the design and conduct of the primary studies as well. We hope in the near future that such guidance is available to investigators aiming to embark on patient preference research.

The major limitation of this work is that we did not conduct a complete systematic review of reviews. We are somewhat limited in our search of the literature since we did not perform the search beyond the PubMed database, did not explode our search terms extensively, and did not search for non-English publications. This could have made us miss some high-quality systematic reviews—although this is not likely to be the case.

## Conclusion

Our survey of the literature demonstrates that there is a strong interest in the healthcare field in conducting research on patient preferences and also on performing systematic reviews of patient preference studies. However, our survey also reveals that there is still room for improvement of the reporting of patient preference studies. Likewise, many methodologies used to perform systematic reviews of these studies need to be tested and refined by researchers as well. In particular, research community should develop research agenda to inform systematic reviewers working on patient preferences of the search for studies, quality assessment of studies, synthesis of studies and reporting of reviews.
